# Estimation of Genetic Parameters for Growth and WSSV Resistance Traits in *Litopenaeus vannamei*

**DOI:** 10.3390/ani14121817

**Published:** 2024-06-18

**Authors:** Juan Sui, Kun Sun, Jie Kong, Jian Tan, Ping Dai, Jiawang Cao, Kun Luo, Sheng Luan, Qun Xing, Xianhong Meng

**Affiliations:** 1 State Key Laboratory of Mariculture Biobreeding and Sustainable Goods, Yellow Sea Fisheries Research Institute, Chinese Academy of Fishery Science, Qingdao 266071, China; suijuan0313@126.com (J.S.); kongjie@ysfri.ac.cn (J.K.); tannjian@163.com (J.T.); daiping54@163.com (P.D.); caobaoxiang401@126.com (J.C.); luokun@ysfri.ac.cn (K.L.); luansheng@ysfri.ac.cn (S.L.); 2 Laboratory for Marine Fisheries Science and Food Production Processes, Qingdao Marine Science and Technology Center, Qingdao 266237, China; 3 College of Fisheries and Life Science, Shanghai Ocean University, Shanghai 201306, China; sunkun6008@163.com; 4 BLUP Aquabreed Co., Ltd., Weifang 261311, China; xingqun527@163.com

**Keywords:** *Litopenaeus vannamei*, whole genome resequencing, ssGBLUP, WSSV resistance, growth

## Abstract

**Simple Summary:**

This study aimed to estimate genetic parameters of growth traits and resistance to white spot syndrome virus in Pacific white shrimp (*Litopenaeus vannamei*). Through controlled virus challenge assays, phenotypic values for five traits were assessed: body weight, overall length, body length, tail length, and post-infection survival time. Three models were utilized, namely pedigree-based best linear unbiased prediction, genomic best linear unbiased prediction, and single-step genomic best linear unbiased prediction. Under the genomic prediction model, the heritability of growth-related traits decreased, whereas the heritability of post-infection survival time increased. Both genomic models significantly enhanced prediction accuracy compared to the pedigree-based model, with the most notable improvement observed in virus resistance. The genetic correlations between growth and WSSV resistance obtained from the three methods were consistently low and negative. These findings provide valuable insights for breeding programs and variety development in *L. vannamei*.

**Abstract:**

The current study aimed to provide a precise assessment of the genetic parameters associated with growth and white spot syndrome virus (WSSV) resistance traits in Pacific white shrimp (*Litopenaeus vannamei*). This was achieved through a controlled WSSV challenge assay and the analysis of phenotypic values of five traits: body weight (BW), overall length (OL), body length (BL), tail length (TL), and survival hour post-infection (HPI). The analysis included test data from a total of 1017 individuals belonging to 20 families, of which 293 individuals underwent whole-genome resequencing, resulting in 18,137,179 high-quality SNP loci being obtained. Three methods, including pedigree-based best linear unbiased prediction (pBLUP), genomic best linear unbiased prediction (GBLUP), and single-step genomic BLUP (ssGBLUP) were utilized. Compared to the pBLUP model, the heritability of growth-related traits obtained from GBLUP and ssGBLUP was lower, whereas the heritability of WSSV resistance was higher. Both the GBLUP and ssGBLUP models significantly enhanced prediction accuracy. Specifically, the GBLUP model improved the prediction accuracy of BW, OL, BL, TL, and HPI by 4.77%, 21.93%, 19.73%, 19.34%, and 63.44%, respectively. Similarly, the ssGBLUP model improved prediction accuracy by 10.07%, 25.44%, 25.72%, 19.34%, and 122.58%, respectively. The WSSV resistance trait demonstrated the most substantial enhancement using both genomic prediction models, followed by body size traits (e.g., OL, BL, and TL), with BW showing the least improvement. Furthermore, the choice of models minimally impacted the assessment of genetic and phenotypic correlations. Genetic correlations among growth traits ranged from 0.767 to 0.999 across models, indicating high levels of positive correlations. Genetic correlations between growth and WSSV resistance traits ranged from (−0.198) to (−0.019), indicating low levels of negative correlations. This study assured significant advantages of the GBLUP and ssGBLUP models over the pBLUP model in the genetic parameter estimation of growth and WSSV resistance in *L. vannamei*, providing a foundation for further breeding programs.

## 1. Introduction

The global production of the Pacific white shrimp (*Litopenaeus vannamei*) reached 6.3 million tons in 2021 [1], cementing its status as one of the preeminent species in shrimp farming worldwide. Primarily, it is distributed along the Pacific coastline of Central and South America, which was introduced to China in 1988. Over three decades of development, its mariculture production surged to 2.09 million tons by 2022, representing close to one-third of the global aquaculture output [2]. However, the shortage of superior germplasm resources severely limits the development of the shrimp aquaculture industry in China. With the continuous expansion of the shrimp farming scale and the adoption of high-density intensive farming models, disease outbreaks are becoming more frequent during the farming process. 

The white spot syndrome virus (WSSV) has been particularly devastating since 1992, often resulting in mortalities exceeding 95% within 7–10 days [3,4,5]. Effective prevention and control measures for this pathogen are currently unavailable. Selective breeding presents a potent strategy for disease control [6,7]. Till now, the National Certification Committee for Aquatic Varieties of China has endorsed twelve novel varieties of *L. vannamei*, such as “Zhongxing No. 1” and “Haixing Nong No. 2”. Nevertheless, growth continues to be the trait of paramount interest to the industry [8,9,10], and 11 out of these 12 varieties exhibit superior growth characteristics. There is an urgent market demand for breeding new varieties that combine both growth rate and WSSV resistance. 

The precise estimation of genetic parameters for objective traits is fundamental to breeding programs, particularly in guiding the formulation of selection indices, breeding stock retention, and mating strategies. Numerous studies have documented the heritability levels of shrimp resistance to WSSV, ranging from 0.03 to 0.53 [3,11,12,13,14]. The genetic correlations between growth and WSSV resistance in different populations ranged from negative to no significant association [15,16,17]. Given the intricate genetic backgrounds of different populations of *L. vannamei*, the assessment of genetic parameters is significantly influenced by the population structure, infection methods, and evaluation methods. Traditional genetic parameter estimation primarily relies on best linear unbiased prediction (pBLUP), which is based on pedigree records. This method, widely utilized in genetic assessment of economic traits in aquaculture species, estimates breeding values through the construction of an A matrix to account for diverse influencing factors [18]. However, due to the risk of introducing pathogens into breeding populations, family selection for enhanced resistance relies on sibling testing, where breeding candidates are shielded from exposure to the pathogen. This approach utilizes only 50% of the additive genetic variance contributed by the between-family component [19,20]. 

Genomic best linear unbiased prediction (GBLUP) is highly regarded for its improved selection accuracy, enabling the evaluation of Mendelian sampling across individuals using genome-wide markers [21]. However, the sequencing of a large number of breeding individuals remains significantly costly. To address this, the single-step genomic best linear unbiased prediction (ssGBLUP) method was devised, which incorporates a pedigree-genomic relationship matrix H by merging the matrix A from the entire pedigree with the genomic relationship matrix G covering all genotyped animals [22,23,24]. During the construction of matrix H, matrix G was scaled to resemble a pedigree relationship matrix by utilizing current allele frequencies, with coefficients ranging from 0 to 1 on the off-diagonals, and diagonal elements being enforced to 1 [25,26]. GBLUP has been used to estimate genetic parameters related to resistance against *Vibrio parahaemolyticus* in *L. vannamei* [27] and for both growth and disease resistance in the banana shrimp *Fenneropenaeus merguiensis* [28]. These studies have demonstrated considerable predictive accuracy in genetic assessments conducted using both GBLUP and ssGBLUP. Additionally, ssGBLUP has been employed to estimate the genetic parameters associated with body weight traits in the giant freshwater prawn *Macrobrachium rosenbergii* [29] and for feed efficiency traits in *L. vannamei* [30]. However, when all animals underwent genotyping, the inclusion of pedigree information in matrix H might diminish the accuracy of realized genomic relationships to some extent due to potential errors in recorded pedigrees [30].

In 2019, we initiated a breeding program for *L. vannamei*, importing eight batches of high-quality germplasm from around the world. These batches included four batches of fast-growing parental shrimp sourced from the United States and five batches of highly resistant parental shrimp sourced from Ecuador. In this study, we conducted growth and WSSV resistance tests on progeny lineages derived from these imported populations. By combining phenotypic, genotypic, and pedigree data, we conducted an estimation of genetic parameters associated with body weight (BW), overall length (OL), body length (BL), total length (TL), and WSSV resistance traits utilizing pBLUP, GBLUP, and ssGBLUP methodologies. This investigation enabled a comparison of various models on genetic parameter estimations and the estimated breeding values (EBV) or genomic EBVs (GEBV). This study can act as a reference for the further development of breeding programs, and an experimental basis for independent breeding of *L. vannamei* varieties with high WSSV resistance and fast growth.

## 2. Materials and Methods

### 2.1. Shrimp and Data Collection

#### 2.1.1. Shrimp

The experimental shrimp used in this study were from the G1 population established in Bangpu Seed Technology Co., Ltd., Weifang City, Shandong Province, China. Eight batches of *L. vannamei* commercial populations were introduced from the United States and Ecuador in 2019, with four batches being highly resistant groups and four batches being rapidly growing groups. Twenty families were produced within six days through mating between selected male and female shrimps in a 1:1 ratio within each group (Table 1), which were tested to be free of Taura Syndrome (TSV), hypodermal and hematopoietic necrosis (IHHNV), acute hepatopancreatic necrosis disease (AHPND), and WSSV. 

#### 2.1.2. Growth Traits Test

The larvae of each family were cultivated in standardized environmental conditions and densities [31]. After 2 months of rearing, the mean body weight reached 5 g. Fifty-five shrimp from each family were randomly chosen and weighed, then placed into 150 L new buckets. The water temperature was gradually reduced by 2–3 °C per day for three days until reaching 20–22 °C, while concurrently adding Vc, glucose, etc., for stress resistance treatment at a concentration of 2.5 ppm. Each individual was wrapped in gauze soaked in seawater, with only the tail exposed. They were tagged individually with different color combinations of “visible implant elastomer” (VIE) at the sixth abdominal segments to distinguish different families. Following tagging, the individuals were immersed in seawater containing 20 ppm povidone-iodine for 30 s for disinfection, then returned to the 150 L buckets for temporary rearing for 3 days, with the temperature gradually increasing to 26–28 °C. All tagged shrimps were cultured in a 50 m^2^ rectangular pond for 2 months. The seawater temperature was controlled at 26 °C to 28 °C and the salinity was at 33‰. All families were fed commercial pellets containing 42% protein, 12% moisture, and 17% crude ash. The feeding amount was adjusted daily based on the growth and remaining feed situation of the shrimp. By the conclusion of the cultivation period, a total of 1040 shrimps were harvested, resulting in a survival rate of 94.55%.

**Figure 1:**
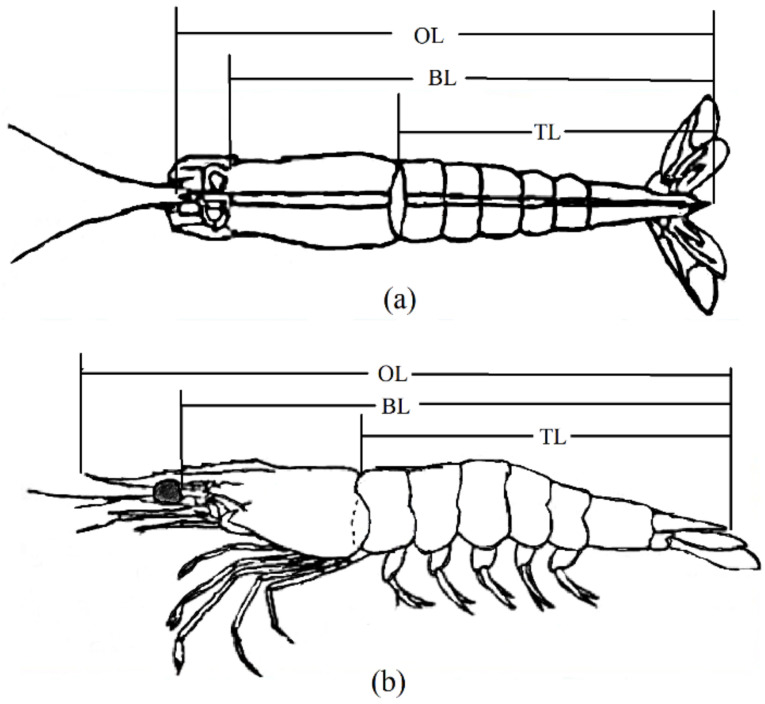
Schematic diagram of morphometric position of *L. vannamei*. (**a**) shows a dorsal view; (**b**) shows a lateral view. OL: overall length; BL: body length; TL: tail length.

The shrimps were transferred to five 10 m^3^ ponds of a trait-testing workshop at the Yellow Sea Fisheries Research Institute. After acclimatizing for 3 days, the excess water of the shrimps was absorbed using absorbent paper and weighed using an electronic balance with a precision of 0.01 g. Each shrimp was simultaneously tagged with an eyestalk ring. The eyestalk tag number, sex, VIE color combination, and body weight of each shrimp were recorded. A digital camera was used to take photographs from the side and back views of the shrimp (each shrimp was photographed twice with a ruler placed beside it). The measured growth traits included overall length (cm, OL), body length (cm, BL), and tail length (cm, TL). Each trait underwent three measurements, and the resultant average value was considered as the final measurement. Water quality control, feeding, and daily management during the transient period remained consistent with the standardized procedures. The morphometric sites for each trait are shown in Figure 1.

#### 2.1.3. WSSV Challenge

After the growth test, the shrimps were acclimatized in the 10 m^3^ ponds for one week. Prior to the WSSV challenge, the age of the shrimps was 180–185 days post-hatch (dph), averaged 183 dph, with an average weight of 16 g. Muscle tissues of shrimps infected with WSSV were used to prepare poisoned bait. The quantification of WSSV load in the muscle was performed using real-time PCR (ABI 7500 real-time PCR system). Muscle tissues were minced and mixed with uninfected muscle tissues and edible red dye to make bait with a concentration of 10^4^ copies of the WSSV genome per milligram (mg). After starvation for 24 h to ensure gastrointestinal evacuation, each shrimp was fed 10 mg bait. Then the shrimps were put back to the pond and observed every hour. The shrimps were provided with formulated diets, portioned into four equal daily rations, constituting a total daily allocation of 5% of the wet weight biomass, and adjusted on a daily basis. Dead individuals were taken out. The hours post infection (h, HPI), color combination, and eyestalk tag number of all testing individuals were recorded. The challenge test lasted for 316 h, until no shrimp deaths occurred within a 24 h period, when the experiment concluded. For each family, an additional 8–10 individuals were prepared, serving as control groups for the test environment. Totally, 1033 individuals were collected for the acquisition of growth and WSSV resistance data (Table 1). Upon examination, all dead individuals exhibited typical features of white spot syndrome (WSS), and five randomly selected shrimps were subjected to real-time PCR testing to confirm WSSV virus infection. Muscle tissues of all testing individuals were collected and stored at −80 °C.

#### 2.1.4. Genotyping

Based on the preliminary experiment, groups R2, R3, and R4 were found to have close kinship. Therefore, 50 individuals were selected from these three populations, whereas 30–70 individuals were randomly chosen from the remaining populations, resulting in a total of 300 individuals for whole-genome sequencing. Genomic DNA was extracted from the muscle tissue using the TIANamp Marine Animals DNA Kit (TIANGEN, Beijing, China). The purity was checked using NanoDrop 1000 (Thermo Scientific, Waltham, MA, USA), integrity was checked using 1% agarose gel electrophoresis, and quantity was checked using Qubit (Thermo Scientific, MA, USA). Whole-genome resequencing was performed using an Illumina HiSeq2500 platform with a sequencing depth of 10× and 125 bp paired end (PE) reads (Beijing Berry Genomics Biotechnology Co., Ltd., Beijing, China).

Raw reads containing more than 10% ambiguous bases (N), displaying poor quality (where nucleotides with a quality value Q ≤ 3 represent over 50% of the read), or harboring adaptors were eliminated. Clean reads from each sample were aligned to the *L. vannamei* genome (*ASM378908v1*) utilizing the Burrows–Wheeler Aligner 0.7 [32] with parameters set as “mem 4-k 32-M” to detect SNPs. Variant calling was executed via GATK’s UnifiedGenotyper. SNPs were subjected to filtration employing GATK’s Variant Filtration with appropriate criteria (-Window 4, -filter “QD < 2.0||FS > 60.0||MQ < 40.0”, -G_filter “GQ < 20”), and those demonstrating segregation distortion or sequencing errors were discarded. Ultimately, 103,745,370 loci were identified. Genotype quality control was conducted using Plink software ver. 1.9 [33], adhering to the subsequent exclusion criteria: SNP call rate < 90%, minor allele frequency < 0.05, Hardy–Weinberg equilibrium with *p*-value < 1 × 10^−6,^ and exclusion of shrimp with a genotype call rate < 90%. Following these steps, 18,137,179 SNPs and 293 animals were retained for further analyses.

### 2.2. Data Analysis

#### 2.2.1. Data and Matrix Construction

The phenotypic data were examined for normal distribution and outliers were removed, resulting in a final dataset of 1017 individuals for statistical analysis. Genetic evaluation for growth and WSSV resistance were conducted using the pBLUP, GBLUP, and ssGBLUP methods. The pBLUP method utilized a relationship matrix A based on pedigree record, which incorporated a two-generation pedigree consisting of 293 individuals and 34 parents. The parental individuals were assumed to be unrelated in the pedigree. The GBLUP method involved the utilization of a G matrix containing genomic information from 293 genotyped individuals. The ssGBLUP method involved the utilization of an H matrix that integrated the pedigree of 1017 individuals and genomic information from a common set of 293 genotyped individuals. 

The A-matrix was constructed using the ASReml-R V4.1 package [34]. The G-matrix based on genotype information and the H-matrix based on combined pedigree information were constructed using the preGSf90 program in BLUPF90 1.70 [35]. Matrix A was allocated weight coefficients of 5%, whereas matrix G was assigned a weight coefficient of 95%.

#### 2.2.2. Variance Components and Heritability Estimates

Variance components for growth and resistance traits were estimated utilizing the ASReml-R V4 package [34] employing the average information constrained maximum likelihood method. The mixed linear models for each trait are provided as follows:

(1) $$y_{ijk}=\mu +bw_{i}+{sex}_{j}+a_{i}+f_{k}+e_{ijk}$$

(2) $$y_{il}=\mu +b{Age}_{i}+{Pond}_{l}+a_{i}+e_{il}$$

where $y_{\;ijk}$ is the observed growth value of the *i*th individual,${\;y}_{il}$ is the observed survival time of the *i*th individual, *b* is the regression coefficient,${\;w}_{i}$ and ${Age}_{i}$ are the mean body weight and age covariates at the time of family tagging, respectively,${\;sex}_{j}$ is the fixed effect of the *j*th gender, ${Pond}_{l}$ is the fixed effects of the *l*th pond,${\;a}_{i}$ is the additive genetic effects of the *i*th individual, a~(0, *M*$\sigma_{a}^{2}$), where *M* is the matrix A, G, or H among individuals, $f_{k}$ is the random effect common to the *k*th full sibling family, *f*~(0, *I*$\sigma_{c}^{2}$), where *I* is an identity matrix, $e_{ijk}$ and $e_{il}$ are the random residual effect of the *i*th individual, *e*~(0, *I*$\sigma_{e}^{2}$). 

Heritability was calculated as follows:

(3) $$h^{2}=\sigma_{a}^{2}/\sigma_{p}^{2}$$

where $h^{2}$ is the heritability, $\sigma_{p}^{2}=\sigma_{a}^{2}+\sigma_{c}^{2}+\sigma_{e}^{2}$ or $\sigma_{p}^{2}=\sigma_{a}^{2}+\sigma_{e}^{2}$, according to the model used.

#### 2.2.3. Z-Test

The *Z*-test was employed to assess the significance of differences in heritability from one or zero [36], as follows:

(4) $$z=\frac{x_{i}-x_{j}}{\sqrt{\left(\sigma_{i}^{2}+\sigma_{j}^{2}\right)}}$$

where $x_{i}$ and $x_{j}$ are the estimates of heritability, $\sigma_{i}$ and $\sigma_{j}$ are their respective standard errors. When testing whether an estimate was significantly different from one, both $x_{j}$ and $\sigma_{j}$ were set to one and zero; when testing whether an estimate was significantly different from zero, both $x_{j}$ and $\sigma_{j}$ were set to zero.

#### 2.2.4. Genetic Correlation

A bivariate animal model was employed to estimate both the phenotypic and genetic correlations between different traits. The bivariate analysis model was identical to the univariate model. The A-matrix, G-matrix, and H-matrix were used in the model to construct the respective additive genetic correlation matrices. 

#### 2.2.5. Prediction Accuracy and Bias

To compare the prediction accuracy of GBLUP and ssGBLUP with pBLUP, a five-fold ten-times cross-validation method was employed. Approximately evenly, 293 sequencing individuals were randomly divided into five groups. One group was randomly selected, and its phenotype data were masked as a validation set, whereas the remaining four groups and untyped individuals’ phenotypes were used as a training set to predict estimated breeding values for the validation population. Ten repetitions of cross-validation were conducted, with predictive accuracy defined as the average Pearson’s correlation coefficient between phenotype values of the validation set and EBV/GEBV, and predictive bias as the regression coefficient of phenotype values against EBV(GEBV). 

A bias of 1 indicates that EBV (GEBV) theoretically provides an unbiased estimate of the true breeding value (BV), whereas a bias of less than or greater than 1 suggests underestimation or overestimation of EBV (GEBV), respectively.

## 3. Results

### 3.1. Descriptive Statistics

Descriptive statistics of BW, OL, BL, TL, and HPI are shown in Table 2. The mean value of BW was 16.21 ± 5.22 g, with a coefficient of variation of 32.20%. The coefficients of variation for other growth traits were lower than that for BW, ranging from 11.55% to 12.48%. The mean value of HPI was 91.53 ± 5.22 h, with the highest coefficient of variation among the test traits, reaching 59.42%. 

### 3.2. Molecular Genetic Correlation Analysis

**Figure 2:**
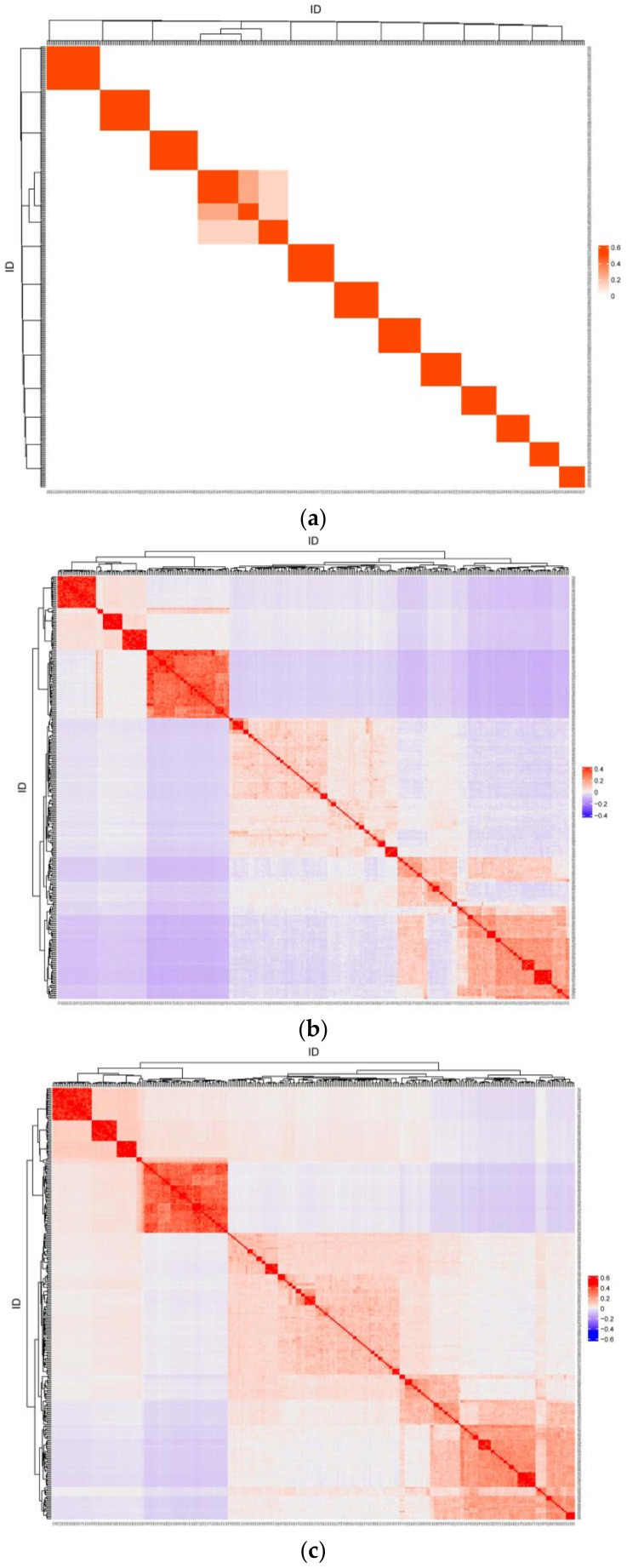
The heatmap of pairwise relatedness coefficients for matrix A (**a**), G (**b**), and H (**c**).

Heatmaps of pairwise relatedness coefficients between individuals for matrices A, G, and H are shown in Figure 2. Only the full-sibling and half-sibling relationships between individuals were reflected based on pedigree kinship, whereas all individuals had specific kinship coefficients with each other based on genotype information. The correlation of kinship coefficients was 0.614 between the lower triangular elements of matrices A and G, 0.649 between matrices A and H, and 0.999 between matrices G and H. 

**Figure 3:**
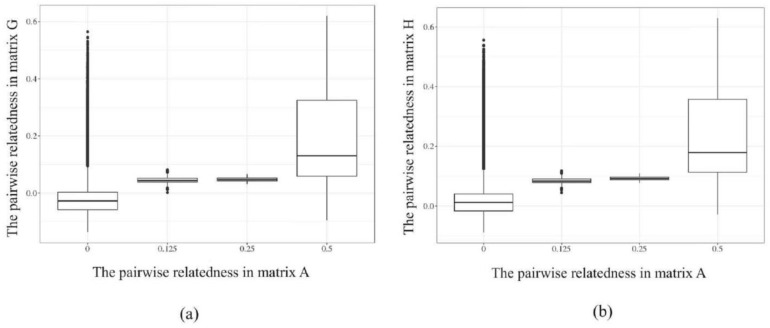
The corresponding relationships in matrix G, H, and matrix A of the sequenced individuals. (**a**) the corresponding relationship between matrix G and A; (**b**) the corresponding relationship between matrix H and A.

The correspondences between matrix G and H with matrix A are shown in Figure 3. When pairwise relatedness coefficients between individuals in matrix A (x-axis) were 0, genomic relatedness among these individuals were higher in G-matrix and H-matrix (y-axis), indicating that the relatedness between individuals was insufficiently accurate relying solely on pedigree information. The same situation was found in genomic relatedness of 0.125, 0.25, and 0.5 relatedness in matrix A. This further demonstrates that matrices G and H, constructed based on molecular markers, can more accurately describe the kinship between individuals compared to matrix A based on a two-generation pedigree.

### 3.3. Heritability

Variance components and heritability estimates of BW, OL, BL, TL, and HPI of 6-month-old shrimp based on matrices A, G, and H are shown in Table 3. According to the classification criteria of Cardellino et al. (1987), the specific heritability values can be divided into four levels; low heritability (0.05–0.15), medium heritability (0.20–0.40), high heritability (0.45–0.60), and very high heritability (>0.65). Based on matrix A, the heritability estimates of growth-related traits (BW, OL, BL, and TL) were from 0.399 ± 0.158 to 0.810 ± 0.103, indicating medium to high levels of heritability. The heritability estimate of HPI was 0.075 ± 0.074, showing no significant difference from 0 (*p* > 0.05). Based on matrix G, the heritability estimates of BW, OL, BL, and TL decreased by 12.10%, 57.19%, 39.28%, and 20.30%, respectively. The estimated heritability of OL did not significantly deviate from 0, whereas other traits exhibited moderate levels of heritability. The heritability of HPI increased by 17.33%, yet still there was no significant difference from 0 (*p* > 0.05). Based on matrix H, the heritabilities of OL, BL, and TL did not significantly differ from 0 (*p* > 0.05). The heritability of BW decreased by 44.07% and 36.38% compared to matrices A and G, respectively, indicating moderate heritability. The heritability of HPI increased by 164% and 125% compared to matrix A and G, respectively, also indicating moderate heritability. For growth-related traits, the estimated heritability decreased from matrices A, and G to H, but increased for WSSV resistance traits.

### 3.4. Genetic Correlation

Genetic and phenotypic correlations among the traits estimated using different methods are shown in Table 4. The genetic correlations among BW, OL, BL, and TL based on pBLUP, GBLUP, and ssGBLUP methods ranged from 0.767 to 0.980, 0.920 to 0.993, and 0.912 to 0.999, respectively. The phenotypic correlations ranged from 0.629 to 0.959, 0.548 to 0.988, and 0.684 to 0.991, indicating medium to high levels of positive correlations. The genetic correlations of HPI with BW, OL, BL, and TL based on pBLUP, GBLUP, and ssGBLUP ranged from −0.198 to −0.081, −0.173 to −0.096, and −0.057 to −0.019, respectively. The phenotypic correlations ranged from −0.219 to −0.162, −0.191 to −0.115, and −0.443 to −0.397, respectively, indicating low to moderate levels of negative correlations. Minor disparities were observed in the genetic and phenotypic correlations of these traits among different methods.

### 3.5. Prediction Accuracy and Bias Analysis

The prediction accuracy and bias of the three methods for BW, OL, BL, TL, and HPI were analyzed using five-fold ten-times cross-validation. The results are shown in Table 5. Compared to pBLUP, GBLUP exhibited increased prediction accuracy for BW, OL, BL, TL, and HPI by 4.77%, 21.93%, 19.73%, 19.34%, and 63.44%, respectively, while reducing bias by 11.88%, 18.49%, 15.94%, 15.14%, and 26.48%, respectively. Moreover, ssGBLUP showed even higher improvements in prediction accuracy, with increases of 10.07%, 25.44%, 25.72%, 19.34%, and 122.58%, and larger reductions in bias by 23.62%, 26.32%, 18.71%, 36.10%, and 50.12%, respectively. When comparing GBLUP to ssGBLUP, the latter demonstrated further enhancements in prediction accuracy by 5.06%, 5.00%, 2.88%, 0%, and 36.18%, respectively. Additionally, ssGBLUP exhibited greater reductions in prediction bias for all traits compared to GBLUP. Among the three methods, ssGBLUP consistently displayed superior prediction accuracy and lower bias, followed by GBLUP, with pBLUP exhibiting the lowest performance in both metrics.

## 4. Discussion

In the selective breeding of *L. vannamei*, growth traits like BL and BW are primary targets, garnering constant attention from breeders. Hence, precise estimation of genetic parameters becomes fundamental for estimating breeding values, formulating breeding programs, and elucidating genetic mechanisms underlying these traits [37]. However, the predominant approach in these studies has been the utilization of the pBLUP method, relying on pedigree information. Numerous investigations, both domestically and internationally, have delved into estimating genetic parameters for growth traits in *L. vannamei*. For example, Xu et al. [38] analyzed phenotypic data from 3240 individuals across 18 half-sibling and 54 full-sibling families, revealing heritability values of 0.460, 0.392, and 0.303 for BW, OL, and BL, respectively. Luan et al. [39] expanded on this with phenotypic data from 19,199 individuals across 130 families, estimating harvest weight heritability between 0.19 and 0.43. Sui et al. [31] further contributed by assessing harvest weight heritability, based on pedigree and phenotypic data from two generations of 24,072 individuals, ranging from 0.278 to 0.423. Hernández-Ruíz et al. [40] reported a harvest weight heritability of 0.24, based on pedigree data from 2002 to 2019 and the phenotypic data of 12,440 individuals from 160 families. In this study, the pBLUP method was combined with the phenotypic data of 1017 individuals from 20 families to assess the heritabilities of the BW, OL, BL, and TL traits in *L. vannamei*, which were found to be 0.810 ± 0.103, 0.654 ± 0.201, 0.690 ± 0.205, and 0.399 ± 0.158, respectively. The heritability of BW estimated based on the A matrix showed a considerable level, which may be significantly influenced by the dataset (population structure) and testing environment. The dataset is relatively small, with only 1014 individuals ultimately included in the analysis. The pedigree depth of test individuals is only two generations, and the parental individuals are sourced extensively and considered unrelated. Variations between families are mainly attributed to genetic differences. During the two-month communal culture period, the stocking density is relatively low (22 ind/m^2^), allowing for significant individual growth differences, with a high coefficient of variation in individual weight (32.2%). These factors have significantly impacted the results of the genetic parameters. A further study could be achieved by increasing the number of test individuals for a more precise parameter. 

Integration of genotypic information using the GBLUP method led to varying degrees of decline in heritabilities for all traits, especially evident in body size traits such as OL, BL, and TL, which declined moderately to high levels, ranging from 0.280 ± 0.154 to 0.419 ± 0.156. These fluctuations in heritabilities could be attributed to factors like population structure, limited family numbers, pedigree documentation, and phenotypic data. Subsequent assessment with the ssGBLUP method, combining genotype and pedigree information, further affected the heritabilities, particularly in BW, whereas the other traits saw minimal changes, resulting in heritability values ranging from 0.195 to 0.453, consistent with previous findings. Notably, body size traits exhibited enhanced prediction accuracy compared to BW. Comparative analysis revealed that both GBLUP and ssGBLUP methods outperformed pBLUP in terms of prediction accuracy and bias reduction for genetic parameter assessment in *L. vannamei*. Moreover, combining pedigree and genotype information in ssGBLUP demonstrated superior prediction accuracy and bias reduction compared to using genotype information alone. Specifically, OL, BL, and BW traits assessed via ssGBLUP displayed 2.88%, 5.00%, and 5.06% higher accuracy, with 9.61%, 3.29%, and 13.32% smaller bias, respectively, compared to GBLUP. Although similar studies on GBLUP and ssGBLUP in *L. vannamei* remain scarce, their successful application in other species underscores their efficacy in genetic parameter assessment for growth traits. Although TL trait accuracy remained unchanged, a notable 24.69% reduction in bias was observed.

WSS has always been a major focus of research on the diseases in *L. vannamei*, and selective breeding for WSSV resistance traits is the next step for breeders. Compared to growth traits, fewer studies have reported on the assessment of genetic parameters for WSSV resistance in *L. vannamei*. For example, Gitterle et al. [15], Caballero-Zamora et al. [16], and Campos-Montes et al. [12] adopted the traditional BLUP method to assess the heritability of WSSV survival traits in *L. vannamei* and found low heritability estimates of 0.03–0.20 for WSSV survival traits. In this study, the heritability of the WSSV resistance trait in *L. vannamei* assessed based on the pBLUP method was not significantly different from 0 (*p* > 0.05). Additionally, it had a prediction accuracy of 0.186 ± 0.058 and a bias of 1.620 ± 0.215, suggesting relatively low prediction accuracy and relatively high bias compared to the growth traits. When genotype information was combined for assessment based on the GBLUP method, the prediction accuracy increased by 63.44%, bias decreased by 26.48%, and the resulting heritability was 0.088 ± 0.081. Furthermore, when both pedigree and genotype information were combined for assessment based on the ssGBLUP method, prediction accuracy increased by 122.58%, bias decreased by 50.12%, and the resulting heritability was 0.198 ± 0.066. Campos-Montes et al. [12] documented the heritabilities concerning the time of death post-WSSV infection and binary survival using GBLUP and ssGBLUP, which ranged from 0.094 to 0.095 and 0.085 to 0.105, respectively, employing a commercial 50 K SNP panel in post-larvae. Discrepancies in heritability estimates may stem from various factors, including the experimental population, population age, and infection methods. The aforementioned studies collectively underscore the efficacy of incorporating genomic information in enhancing the precision of heritability estimates. The integration of genomic data is anticipated to refine the estimation of variance components by providing a more precise assessment of parentage relationships, thereby facilitating a clearer demarcation between additive and residual genetic variance [30,41]. Similar results were also observed in genomic evaluations of disease-resistance traits in other species. Robledo et al. [42] used GBLUP to assess resistance to amoebic gill disease in Atlantic salmon, and their results showed higher accuracy for GBLUP (0.62) than for pBLUP (0.51). Moreover, the ssGBLUP method was found to be superior to the GBLUP method. Several reports have explored the use of GBLUP and ssGBLUP to assess genetic parameters for disease resistance traits in aquatic animals. Sukhavachana et al. [43] also used the GBLUP method to study the resistance of *Streptococcus agalactiae* in hybrid red tilapia, with GBLUP accuracy at 0.25, compared to pBLUP at 0.15. Tsai et al. [44] assessed the resistance to sea lice in Atlantic salmon under different SNP densities (0.5–33 k). Vallejo et al. [45] used the ssGBLUP method to assess genetic parameters of resistance to bacterial cold-water disease in rainbow trout, improving prediction accuracy by 83.3–85.3%. Additionally, Lu et al. [36] used the ssGBLUP method to assess genetic parameters of Edwardsiellosis resistance in Japanese flounder, achieving a final prediction accuracy of 0.65 for ssGBLUP, compared to 0.54 for pBLUP. Yoshida et al. [46] assessed genetic parameters of resistance against salmonid rickettsial syndrome in rainbow trout under different marker densities (0.5–27 k), showing improved prediction accuracy by 21–37% using the GBLUP method and 23–40% using the ssGBLUP method. Our results align with these findings, indicating that the GBLUP and ssGBLUP methods yield higher prediction accuracy than the pBLUP method for assessing genetic parameters for disease resistance traits. One issue worth noting is that marker density and the number of sequenced individuals may be critical factors affecting the accuracy of assessments. However, excessively high marker density and a large number of individuals can also increase sequencing costs. Choosing an appropriate sequencing strategy is a matter that requires consideration.

It has been demonstrated that there exists a strong positive correlation between growth traits in *L. vannamei* (r_g_ > 0.95), suggesting a close linkage between the genes governing body weight (BW) and body length (BL). Consequently, simultaneous selection for BW alongside BL can yield more favorable outcomes [47]. In this study, we estimated the genetic and phenotypic correlations among BL, BW, OL, and TL traits using various assessment models that incorporate genotypic information. Our findings echoed previous studies, indicating minimal effects of the models on genetic correlations and phenotypes. Additionally, we investigated the genetic and phenotypic correlations between growth and WSSV resistance traits using different models. Our results revealed that the models had a relatively minor impact, with growth and WSSV traits exhibiting genetic correlations ranging from −0.198 to −0.019 and phenotypic correlations from −0.443 to −0.115. Genetic correlation studies between growth and viral disease resistance often show negative associations in aquatic animals. In shrimp, Argue et al. [48] reported a genetic correlation of −0.46 between growth and Taura syndrome resistance. Fu et al. [17] found genetic correlations ranging from −0.034 to −0.573 between growth and WSSV resistance. In fish, Bangera et al. [49] reported genetic correlations of −0.25 and 0.032 between growth rate and vibriosis and viral nervous necrosis, respectively, and both were not significantly different from zero. Cock et al. [50] proposed that resistance genes may encounter antagonistic selective pressures, leading to an equilibrium frequency that falls short of complete fixation. Given the prolific reproductive capacity of shrimp, extensive collection of diverse populations allows for the construction of numerous families for resistance testing, facilitating the identification of resistance sources even at low frequencies. Anyway, it is imperative to incorporate economic weighting coefficients or percentage allocations for both growth traits and WSSV resistance traits in *L. vannamei* breeding programs. Developing a multi-trait composite selection index can facilitate the assessment and selection of high-quality broodstock.

## 5. Conclusions

This study demonstrated that the heritability of growth-related traits obtained from GBLUP and ssGBLUP was lower than that obtained from pBLUP, whereas the heritability of WSSV resistance was higher than that obtained from pBLUP. The predictive accuracy of growth traits and WSSV resistance obtained from the GBLUP and ssGBLUP methods was higher than that from the pBLUP method, with lower predictive biases than pBLUP. The genetic correlations of growth and WSSV resistance obtained from the three methods were low negative. This study’s findings offer methodologies and valuable insights for assessing genetic parameters in *L. vannamei*, laying the groundwork for future breeding programs and variety development.

## Figures and Tables

**Table 1:** Number of families and analyzed individuals by batch and group.

Batch	Group	Family No.	Phenotyped Individual No.	Genotyped Individual No.
1	R1	2	98	49
2	R2	5	262	1
3	R3	3	162	49
4	R4	1	50	0
5	G1	3	154	28
6	G2	3	151	37
7	G3	2	105	67
8	G4	1	51	62
Total	/	20	1033	293

Note: R: highly resistant group; G: rapidly growing group.

**Table 2:** Descriptive statistics of phenotypic values of growth and WSSV resistance.

Parameter	Mean	Max	Min	SD	CV
BW (g)	16.21	31.95	6.64	5.22	32.20%
OL (cm)	14.54	20.90	10.16	1.68	11.55%
BL (cm)	11.32	16.10	7.26	1.40	12.37%
TL (cm)	8.09	11.38	2.70	1.01	12.48%
HPI (h)	91.53	316.00	40.00	54.39	59.42%

Note: BW: body weight, OL: overall length, TL: tail length, BL: body length, HPI: hours post-infection, SD: standard deviation, CV: coefficient of variation.

**Table 3:** Variance components and heritability of BW, OL, BL, TL, and HPI of 6-month-old shrimp based on different methods.

Matrix	Traits	$\sigma_{p}^{2}$	$\sigma_{a}^{2}$	C	$h^{2}$
A-matrix	BW	13.59	11.073	0.941	0.810 ± 0.103 ^a^
	OL	1.749	1.144	1.47 × 10^−5^	0.654 ± 0.201
	TL	0.747	0.298	6.20 × 10^−7^	0.399 ± 0.158
	BL	0.747	0.298	6.20 × 10^−7^	0.690 ± 0.205
	HPI	3990.088	301.038	\	0.075 ± 0.074 ^b^
G-matrix	BW	8.976	6.395	1.095	0.712 ± 0.172
	OL	1.474	0.413	0.216	0.280 ± 0.154 ^b^
	TL	0.654	0.208	0.006	0.318 ± 0.128
	BL	0.654	0.208	0.006	0.419 ± 0.156
	HPI	3972.164	348.929	\	0.088 ± 0.081 ^b^
H-matrix	BW	13.482	6.113	3.491	0.453 ± 0.169
	OL	1.800	0.351	0.391	0.195 ± 0.146 ^b^
	TL	0.647	0.167	0.037	0.258 ± 0.149 ^b^
	BL	0.647	0.167	0.037	0.316 ± 0.162 ^b^
	HPI	11,027.88	2180.745	\	0.198 ± 0.066

Note: BW: body weight, OL: overall length, TL: tail length, BL: body length, HPI: hours post-infection, $\sigma_{p}^{2}$: phenotypic variance, $\sigma_{a}^{2}$: additive genetic variance, C: common environmental effects, $h^{2}$: heritability. ^a^: estimate is not significantly different from 1 (*p* > 0.05). ^b^: estimate is not significantly different from 0 (*p* > 0.05).

**Table 4:** Genetic and phenotypic correlations of BW, OL, BL, TL, and HPI based on different methods.

Method	Traits	OL	TL	BL	BW	HPI
pBLUP	OL	1	0.767	0.909	0.980	−0.081
	TL	0.959	1	0.946	0.972	−0.163
	BL	0.988	0.831	1	0.970	−0.198
	BW	0.741	0.629	0.860	1	−0.162
	HPI	−0.216	−0.162	−0.219	−0.163	1
GBLUP	OL	1	0.978	0.920	0.955	−0.170
	TL	0.958	1	0.986	0.971	−0.106
	BL	0.988	0.806	1	0.993	−0.173
	BW	0.798	0.548	0.838	1	−0.096
	HPI	−0.186	−0.124	−0.191	−0.115	1
ssGBLUP	OL	1	0.974	0.912	0.990	−0.019
	TL	0.981	1	0.983	0.999	−0.032
	BL	0.991	0.909	1	0.982	−0.057
	BW	0.864	0.684	0.871	1	−0.057
	HPI	−0.412	−0.397	−0.443	−0.443	1

Note: the value in the lower triangle is the phenotypic correlation between traits, and the value in the upper triangle is the genetic correlation between traits. OL: overall length, TL: tail length, BL: body length, BW: body weight, HPI: hours post-infection.

**Table 5:** Prediction accuracy and bias of each trait under different methods.

Traits	pBLUP		GBLUP		ssGBLUP	
	Accuracy	Bias	Accuracy	Bias	Accuracy	Bias
BW	0.566 ± 0.029	1.431 ± 0.173	0.593 ± 0.041	1.261 ± 0.097	0.623 ± 0.027	1.093 ± 0.058
OL	0.456 ± 0.043	1.493 ± 0.119	0.556 ± 0.025	1.217 ± 0.035	0.572 ± 0.017	1.100 ± 0.147
TL	0.424 ± 0.054	1.532 ± 0.186	0.506 ± 0.018	1.300 ± 0.060	0.506 ± 0.054	0.979 ± 0.088
BL	0.451 ± 0.025	1.336 ± 0.056	0.540 ± 0.017	1.123 ± 0.209	0.567 ± 0.013	1.086 ± 0.101
HPI	0.186 ± 0.058	1.620 ± 0.215	0.304 ± 0.040	1.191 ± 0.324	0.414 ± 0.025	0.808 ± 0.187

Notes: BW: body weight; OL: overall length; TL: tail length; BL: body length. HPI: hours post-infection.

## Data Availability

The original contributions presented in the study are included in the article, further inquiries can be directed to the corresponding author.
